# Kinematic Sub-Populations in Bull Spermatozoa: A Comparison of Classical and Bayesian Approaches

**DOI:** 10.3390/biology9060138

**Published:** 2020-06-26

**Authors:** Luis Víquez, Vinicio Barquero, Carles Soler, Eduardo R.S. Roldan, Anthony Valverde

**Affiliations:** 1 Costa Rica Institute of Technology, School of Agronomy, San Carlos Campus, 223-21002 Alajuela, Costa Rica; luisga.viquez@gmail.com (L.V.); vinicio1196@gmail.com (V.B.); 2 Department of Cellular Biology, Functional Biology and Physical Anthropology, University of Valencia, Campus Burjassot, C/Dr Moliner, 50, 46100 Burjassot, Spain; carles.soler@uv.es; 3 Proiser R+D, Av. Catedrático Agustín Escardino, 9, Building 3 (CUE), Floor 1, 46980 Paterna, Spain; 4 Department of Biodiversity and Evolutionary Biology, Museo Nacional de Ciencias Naturales (CSIC), 28006 Madrid, Spain; roldane@mncn.csic.es

**Keywords:** spermatozoa, bull, cluster, kinematics, motility, CASA

## Abstract

The ejaculate is heterogenous and sperm sub-populations with different kinematic patterns can be identified in various species. Nevertheless, although these sub-populations are statistically well defined, the statistical differences are not always relevant. The aim of the present study was to characterize kinematic sub-populations in sperm from two bovine species, and diluted with different commercial extenders, and to determine the statistical relevance of sub-populations through Bayesian analysis. Semen from 10 bulls was evaluated after thawing. An ISAS^®^v1 computer-assisted sperm analysis (CASA)-Mot system was employed with an image acquisition rate of 50 Hz and ISAS^®^D4C20 counting chambers. Sub-populations of motile spermatozoa were characterized using multivariate procedures such as principal components (PCs) analysis and clustering methods (*k-means* model). Four different sperm sub-populations were identified from three PCs that involved progressiveness, velocity, and cell undulatory movement. The proportions of the different sperm sub-populations varied with the extender used and in the two species. Despite a statistical difference (*p <* 0.05) between extenders, the Bayesian analysis confirmed that only one of them (Triladyl^®^) presented relevant differences in kinematic patterns when compared with Tris-EY and OptiXcell^®^. Extenders differed in the proportion of sperm cells in each of the kinematic sub-populations. Similar patterns were identified in *Bos taurus* and *Bos indicus*. Bayesian results indicate that sub-populations SP_1_, SP_2_, and SP_3_ were different for PC criteria and these differences were relevant. For velocity, linearity, and progressiveness, the SP_4_ did not show a relevant difference regarding the other sperm sub-populations. The classical approach of clustering or sperm subpopulation thus may not have a direct biological meaning. Therefore, the biological relevance of sperm sub-populations needs to be reevaluated.

## 1. Introduction

Fertility in cattle, as in other species, is a key determinant of productivity and, thus, understanding factors that affect fertility in beef and dairy herds is of utmost importance [1]. Most of the artificial insemination performed in dairy cattle is done with frozen-thawed semen, whereas for beef cattle it is mainly used in genetic stations for selection and in developed farms. Although it is well known that sire fertility is related to sperm motility and kinematic patterns [2] the effects of semen quality on reproductive efficiency in cattle are not yet fully understood [3]. Cryopreservation causes damages to spermatozoa resulting in lower fertilizing capacity [4]. Individual variation in semen freezability exists in most domestic species, including bulls [5] and pigs [6], and such variation may be explained, at least in part, by the patterns of motile sperm sub-populations (SPs) in the ejaculate [7].

The intrinsic variability of semen samples, as well as individual variation or differences arising as a result of treatments, can be studied by using computer-assisted sperm analysis (CASA)-Mot systems that allow for the generation of huge datasets consisting of kinematics trajectories from thousands of spermatozoa [8]. CASA systems have evolved rapidly during the last decade due to major innovations in technology [9] such as increases in computational memory [10,11], use of 3D technology [12], considerations of the effect of frame rate capture [13,14,15,16], improvements in camera acquisition [17], or new approaches for sperm cell tracking [11,18,19]. Current CASA-Mot systems can be used to analyze individual sperm kinematics more accurately and this information can be submitted to a multivariate procedure, such as cluster analysis, for an overview of distinct sperm patterns grouped into SPs or clusters [20].

Cluster analysis divides data into groups (clusters) that are meaningful, useful, or both. If the goal is to obtain meaningful groups, then the cluster should capture the natural structure of the data. In some cases, however, cluster analysis is only a useful starting point for other purposes, such as data summarization. Whether for understanding or utility, cluster analysis has long played an important role in a wide variety of fields [21]. In many areas of biology, scientists have devoted considerable effort to generate groups with hierarchical classification criteria. More recently, clustering has been employed to analyze the large amount of information that is obtained by using CASA-Mot systems. For example, clustering has been used to find groups of spermatozoa that have similar patterns. Thus, several studies in many species have identified the existence of different sperm SPs, defined by specific kinematic patterns obtained from CASA-Mot systems [13,22,23,24,25,26,27,28,29,30,31,32]

The aim of the present study was to assess whether statistical differences in kinematic patterns of bull sperm SPs were relevant according to Bayesian analyses. In addition, we examined possible differences in sperm SPs in bull semen of *Bos taurus* and *Bos indicus* diluted with different extenders.

## 2. Materials and Methods

This study was performed following ethical principles and with the approval of the Committee of Centro de Investigación y Desarrollo de la Agricultura Sostenible para el Trópico Húmedo at the Costa Rica Institute of Technology (CIDASTH-ITCR) according to Section 01/2019, article 1.0, DAGSC-074-2019.

### 2.1. Semen Collection and Processing

Bulls used in this study were of various breeds of *Bos taurus* and *Bos indicus*, with an average age of 5.7 ± 2.8 years. Straws of frozen semen were supplied by Avance Genético S.A. (Zapote, Costa Rica). Semen was collected with an artificial vagina, under a collection program of two ejaculates per week, which had no apparent changes in animal health or semen quality throughout the semen collection period. All bulls were routinely used for semen collection for commercial purposes. Data from 20 ejaculates obtained from 10 randomly selected bulls were employed in this study. The bulls had passed a standard breeding soundness evaluation, and had produced sperm with acceptable post-thaw characteristics (progressive motile sperm >60%) and fertility (non-return rate >65%).

Within 5 to 10 min of collection, the semen samples were assessed for volume, by using a conical tube graduated at 0.1 mL, gross motility, by placing 20 µL of fresh raw semen on a prewarmed slide at 37 °C, and concentration, employing a bovine photometer Accucell (IMV, L’Aigle, France) at 530 nm wavelength. The raw semen was diluted with Tris-citric acid-egg yolk extender (Tris-EY) and two commercial egg yolk extenders (OptiXcell ^®^-IMV, L’Aigle, France; Triladyl^®^, Minitube, Tiefenbach, Germany) to give a final live sperm concentration of 25 × 10^6^ cells/straw. Diluted semen was cooled slowly to 4 °C at a linear rate of −0.3°C min^−1^ in a refrigerator. After cooling, equilibration took place over a period of 4–5 h at the same temperature.

Tris-EY extender was prepared by using 1.36 g citric acid (Fisher Scientific, UK), 2.42 g Tris (hydroxymethyl) aminomethane (Research Organics, USA), 1.0 g glucose (Scharlau, Spain), 14.0 mL glycerol (Merck, Germany), 20% egg yolk in 80 mL distilled water. Antibiotics, namely gentamicin sulfate (100 mg/mL; Sigma Chemical Co. St. Louis, MO, USA) was added to the Tris-EY extender. The commercial extenders were used following the manufacturer’s instructions.

Semen was packaged in 0.25 mL straws with an automatic filling and sealing machine (MRS 1, IMV Technologies, L’Aigle, France) and frozen by a programmable freezer, (Digitcool 5300, IMV, L’Aigle, France) with the following curve: 4 °C to −10 °C at 5 °C min^−1^, −10 °C to −100 °C at 40 °C min^−1^, −110 °C to −140 °C at 20 °C min^−1^, and then plunged into liquid nitrogen for storage. All samples were coded in such a way that the technician who performed the kinematics analysis did not know the number of the bull, the number of the ejaculate, or which ejaculate belonged to a particular bull.

### 2.2. Assessment of Sperm Variables

The cryopreserved semen samples were thawed in a water bath (37 °C, 60 s) and examined immediately after thawing. Disposable counting chambers for the analysis of motility and kinematics variables (ISAS^®^D4C20, Proiser R+D, S.L., Paterna, Spain) were used after pre-warming them to 37 °C. After thorough mixing of the diluted semen samples, 2.7 µL of diluted semen were placed in the counting chamber tracks by capillarity. Analyses were conducted with the CASA-Mot system ISAS^®^v1 (Integrated Semen Analysis System, Proiser R+D, Paterna, Spain) fitted with a video-camera (Proiser 782M, Proiser R+D), a frame rate of 50 frames per second (fps) and a final resolution of 768 × 576 pixels. The camera was attached to a microscope UB203 (UOP/Proiser R+D) with a 1X eyepiece and a 10× negative-phase contrast objective (AN 0.25), and an integrated heated stage maintained at 37 ± 0.5 °C.

### 2.3. Computerized Kinematic Analysis

CASA analyses were performed recording seven microscope fields with a total of at least 600 cells per sample. The CASA-Mot variables assessed in this study included: straight-line velocity (VSL, µm·s^−1^), corresponding to the straight line from the beginning to the end of the track; curvilinear velocity (VCL, µm·s^−1^), measured over the actual point-to-point track followed by the cell; average path velocity (VAP, µm·s^−1^), the average velocity over the smoothed cell path; amplitude of lateral head displacement (ALH, µm), defined as the maximum of the measured width of the head oscillation as the sperm swims; beat-cross frequency (BCF, Hz), defined as the frequency with which the actual track crosses the smoothed track in either direction; motility (%), the percentage of total motile cells and progressive motility (%), corresponding to spermatozoa swimming rapidly forward in a straight line (assessed as straightness index ≥45%; VAP ≥25 µm·s^−1^). Three progression ratios, expressed as percentages, were calculated from the velocity measurements described above: linearity of forward progression (LIN = VSL/VCL·100), straightness (STR = VSL/VAP·100), and wobble (WOB = VAP/VCL·100).

### 2.4. Statistical Analysis

The data obtained for the analysis of all sperm parameters were first assessed for normality and homoscedasticity by using Shapiro-Wilks and Levene tests. A normal probability plot was used to assess normal distribution. Multivariate procedures were performed to identify sperm SPs from the set of sperm motility data. All the values for kinematic variables were standardized to avoid any scale effect.

#### 2.4.1. Multivariate Analysis

The first process carried out was a principal component analysis (PCA) of these data to derive a small number of linear combinations that still retained as much information as possible from the original variables. The number of principal components (PC) used in the next part of the analysis was determined from the Kaiser criterion, namely selecting only those with an eigenvalue (variance extracted of each PC) >1. Furthermore, Bartlett’s sphericity test and the KMO (Kaiser-Meyer-Olkin) were performed [33]. As a rotation method, the varimax method with Kaiser normalization was used [34].

The second process was conducted to perform a non-hierarchical analysis with the *k-means* model that uses Euclidean distances from the quantitative variables after standardization of these data, so the cluster centers were the means of the observations assigned to each cluster [35]. The multivariate *k-means* cluster analysis was made to classify the spermatozoa into a reduced number of SPs (clusters) according to their kinematic variables. In the final process, to determine the optimal number of clusters, the final centroids were clustered hierarchically using the Ward method [36]. Thus, the clustering procedure enables for the identification of sperm SPs because each cluster contributed to a final cluster formed by the spermatozoa linked to the centroids. The analysis of variance (ANOVA) and χ^2^-test procedures were applied to evaluate statistical differences in the distributions of observations (individual spermatozoa) within SPs and then a generalized linear model (GLM) procedure was used to determine the effects of the breed and extender type on the mean kinematic variable values defining the different sperm SPs (i.e., the cluster centers). Differences between means were analyzed by Bonferroni test. Results are presented as mean ± standard error of the mean (SEM). Statistical significance was considered at *p* < 0.05. All data were analyzed using IBM SPSS package, version 23.0 for Windows (SPSS Inc., Chicago, IL, USA).

#### 2.4.2. Bayesian Analysis

Differences in sperm kinematic patterns were estimated with a model including the effect of extender and species as a permanent effect. Male number was included as a random effect. All analyses were performed using Bayesian methodology. The posterior mean of the difference between extenders (*D*), the highest posterior density region at 95% (HPD_95%_) and the probability of the difference being positive when *D* > 0 or negative when *D* < 0 (*P*_0_) were calculated. Bounded uniform priors were used for all effects. Residuals were a priori normally distributed with mean 0 and variance σ^2^_e_. We considered one third of the standard deviation (s.d.) of a trait as a relevant value (R), and we also calculated the probability of relevance (*P_R_*; i.e., the probability of the difference being greater than R when *D* > 0 or lower than R when *D* < 0). The priors for the variances were also bounded uniform. Features of the marginal posterior distributions for all unknowns were estimated using Gibbs sampling. Convergence was tested using the *Z* criterion of Geweke [37] and Monte Carlo sampling errors were computed using time series procedures described in [38]. The Rabbit program, developed by the Institute for Animal Science and Technology (Valencia, Spain), was used for all procedures.

## 3. Results

### 3.1. Overall Motility Parameters

The mean (± SEM) total motility (%) of samples cryopreserved in the different extenders was 51.50 ± 8.38 (OptiXcell^®^), 53.83 ± 3.78 (Triladyl^®^) and 58.38 ± 4.63 (Tris-EY) with an overall range of 18–79%. The progressive motility of sperm (%) for OptiXcell^®^, Triladyl^®^ and Tris-EY was, respectively, 36.33 ± 6.81, 40.83 ± 2.44 and 37.75 ± 4.39. Average total motility (%, mean ± SEM) for *Bos taurus* and *Bos indicus* bulls was 58.33 ± 3.05 and 49.88 ± 6.45, respectively. The progressive motility (%) for the two species was 41.92 ± 2.09 (*Bos taurus*) and 32.75 ± 5.56 (*Bos indicus*).

### 3.2. Subpopulation Structure

**Figure 1:**
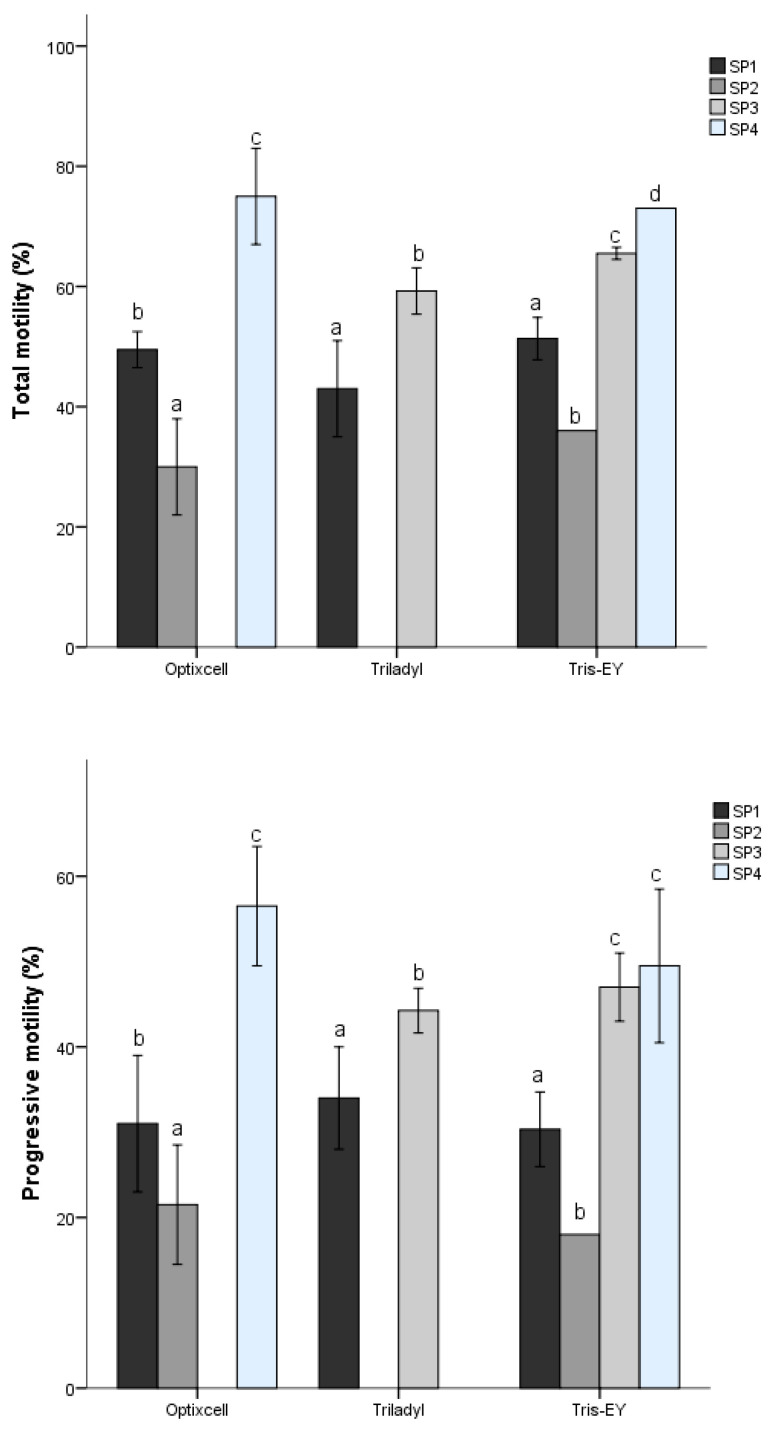
Distribution of total motility and progressiveness of sperm within sub-populations (SPs) between extenders. Different letters (a–d) indicate differences within SPs between extenders. *p* < 0.05.

After multivariate cluster analysis (*k-means* model), four motile sperm SPs with different kinematic patterns were identified. There was extender effect (*p <* 0.05) on SPs distribution of the percentages motile- and progressively motile spermatozoa (Figure 1). Summary data for kinematics of the SPs are presented in Table 1. They can be summarized as follows: subpopulation 1 (SP_1_) included sperm with medium velocity (intermediate values of VCL, VSL, and VAP) and linear and progressive motility (inferred from intermediate LIN and STR values). This population included 20.02% of the total motile sperm. Subpopulation 2 (SP_2_) contained active, fast, linear, and progressive sperm, as indicated by the greater value of VCL, VSL, and VAP, together with the lower values of LIN and STR, and intermediate value of BCF. Moreover, the ALH value was the lowest seen in all SPs, indicating movement with few undulatory characteristics. About 28.29% of the total motile sperm were assigned to this subpopulation. Subpopulation 3 (SP_3_) included 19.14% of the total sperm and was represented by slow motile sperm with the lowest velocity but non-undulatory as revealed by values of VAP, VCL, VSL, and BCF. This population had lesser progressive motility than the other SPs as indicated by LIN and ALH values. Subpopulation 4 (SP_4_) contained 32.54% of the total motile sperm population, and these cells had the highest motility and progressive motility, but movements were undulatory, as indicated by the high values of VCL, VSL, VAP, ALH, and BCF, together with the highest values of LIN, STR, and WOB.

### 3.3. Sperm Kinematics within Sub-Populations for Different Extenders

Results from PCA revealed three PCs in all extenders (Table 2). PC1 in OptiXcell^®^, Triladyl^®^ and Tris-EY was referred to as “progressiveness” and was represented by LIN, STR, and WOB. The larger eigenvectors corresponded to LIN (OptiXcell^®^: 0.931; Triladyl^®^: 0.977), or WOB (Tris-EY: 0.942). PC2 showed a “velocity” component, with high values for VCL and ALH. The greater effect was due to VCL in all extenders (OptiXcell^®^: 0.960; Triladyl^®^: 0.915; Tris-EY: 0.899). PC3, represented by STR and BCF, was named “undulatory movement”. It mainly related to BCF (OptiXcell^®^: 0.801; Triladyl^®^: 0.952), or STR (Tris-EY: 0.844). These results indicate that sperm progressiveness has a relatively greater effect on the total variance than the other variables (Table 2).

There were differences between sperm SPs within each extender (*p* < 0.05) with VCL and LIN exhibiting the highest differences between the sub-populations. The SP distribution was not the same for each extender. The highest VCL values were seen in SP_2_ for OptiXcell^®^ (165.50 ± 1.20 µm·s^−1^), and SP_3_ for Triladyl^®^ (168.80 ± 1.27 µm·s^−1^) and Tris-EY (168.04 ± 0.97 µm·s^−1^). For LIN, the highest values were in SP_2_ in OptiXcell^®^ (87.16 ± 0.53%), SP_1_ in Triladyl^®^ (85.91 ± 0.61%) and SP_2_ in Tris-EY (88.45 ± 0.57%). When each sperm subpopulation was compared between extenders, kinematics differences (*p* < 0.05) were found. The variables with the greatest differences between extenders for all SPs were VCL, LIN and WOB (Table 3).

### 3.4. Bayesian Analysis of Sperm Sub-Populations

Bayesian analysis of the data showed that VCL, VAP, LIN and BCF exhibited relevant differences between Tris-EY and Triladyl^®^, that VCL and VAP presented relevant differences between Tris-EY and OptiXcell^®^, and that only WOB showed relevant differences between Triladyl ^®^ and OptiXcell^®^. In the other cases, despite differences being significant (*p* < 0.05) between all extenders (VSL) or at least two extenders (LIN, WOB), they were not relevant (Table 4).

Bayesian analysis of the data (posterior distribution) when comparing *Bos taurus* and *Bos indicus* showed that even when differences (*p* < 0.05) were identified ensuing frequentist statistics (confidence distribution), these differences between kinematic variables were considered non-relevant (Table 5).

When the relevance of differences between sperm sub-populations was analyzed, the Bayesian analyses of data showed relevant differences between the following comparisons of SPs: SP_1_−SP_2_ (for VSL and LIN); SP_1_−SP_3_ (all kinematic variables except VCL, WOB and BCF); SP_1_−SP_4_ (for VSL); SP_2_−SP_3_ (all variables except VSL); SP_2_−SP_4_ (for STR, WOB, ALH, and BCF). Thus, the sub-populations SP_1_, SP_2_ and SP_3_ showed differences (*p* < 0.05) for most of the kinematic variables when using the classical approach, and these differences were also revealed by the Bayesian approach. On the other hand, the SP_3_−SP_4_ comparison did not show relevant differences for any kinematic variable. Even though subpopulation SP_4_ showed differences regarding SP_2_ or SP_3_, such differences should be regarded as non-relevant for VCL, VSL, VAP, LIN, WOB, or BCF (Table 6).

### 3.5. Distribution of the Sperm Subpopulation

Analysis of the proportion of sperm cells in each subpopulation for the three extenders revealed differences between extenders and SPs for each extender. The sperm SPs were unevenly distributed for each extender. The sperm sub-populations with highest number of cells were associated with a specific extender [(OptiXcell^®^: SP_2_, 36.06%; Triladyl^®^: SP_4_, 35.62%; Tris-EY: SP_4_, 34.66%). On the other hand, sub-populations with lower percentages of cells in Tris-EY and OptiXcell^®^ were associated with SP_1_ (12.00%) and SP_3_ (13.60%), respectively (Table 7).

## 4. Discussion

Cryopreservation is frequently used in animal production and there are several sources of variation in the success of semen survival during this procedure [4]. During freezing and thawing variation relates, for instance, to the effect of extender, which can influence the kinematic patterns of spermatozoa. Breed and inter-individual variation post-thawing can relate to differences in plasma membrane properties in response to freezing and thawing. Cryopreservation can result in alterations in the semen quality due to increases of oxidative stress which in turn can generate changes in sperm motility and kinematic patterns, DNA fragmentation, alterations in timing of capacitation or the acrosome reaction [39]. The evaluation of these changes is very difficult, if not impossible, when employing a classical analysis of semen quality and, thus, the use of CASA technology is important for the improvement of sperm quality assessments [2,40,41,42]. CASA-Mot systems have demonstrated greater accuracy in motility evaluation compared with subjective methods [10]. Moreover, these systems provide abundant information on many kinematic variables for each spermatozoon [8,43] that can be used as a basis for the analysis of the heterogeneity of sperm populations. However, a strategy used by artificial insemination centers is to compensate damage to spermatozoa by increasing the number of motile sperm cells in the insemination doses, and this can mask the relevance of sperm SPs.

In bulls, it has been suggested that sperm SPs with greater percentages of fast yet nonlinear spermatozoa seem to have greater fertilization capacity [25]. Other work indicates that fast and nonlinear sperm movements are in fact specific of hyperactivated spermatozoa [44]. Results of other studies suggest that the sperm SP containing fast and linear spermatozoa is the one with a higher likelihood of sperm-oocyte interaction [28]. Despite these attempts at linking SP structure and function, including sperm fertility, the abundant data provided by CASA-Mot systems for multivariate procedures, and the resulting SP, are still insufficient to categorize an ejaculate in relation to its quality. Moreover, the classification of SPs through classical clustering procedures remains uncertain and even controversial. This relates to the observation that the multivariate procedures of clustering will find several kinematics patterns in the data set, even if there are no natural clusters in the data [45].

The multivariate approach of PCs and cluster analysis conducted for several species underscores that ejaculates are nevertheless heterogeneous in that they contain spermatozoa with different motility and kinematic patterns [17,22,31,46,47,48,49,50,51,52,53]. In the present study, we identified four different sperm SPs described from three PCs that represented velocity, progressiveness, and cell undulatory movement. The proportions of the different SPs varied with the extender used and among species. The cluster of fast and linear sperm did not follow a consistent pattern between SPs; in any case, the samples diluted with Triladyl^®^ showed the greatest kinematic activity. In our study, despite differences (*p* < 0.05) between extenders, the Bayesian analysis revealed that only Triladyl^®^ presented relevant differences in comparison to Tris-EY and OptiXcell^®^ for these kinematic patterns. Our results indicate that subpopulation distribution was also different between and within bovine species. In contrast, other studies have indicated that the sperm subpopulation distribution was not different within species [5]. On the other hand, for the two bull species (*Bos taurus, Bos indicus*), the overall distribution of sperm subpopulation structure showed little variation among individuals.

The application of biostatistical analysis tools to the study of sperm kinematic variables revealed the existence of sperm SPs in the ejaculate [9,13,23,54,55,56,57,58,59], but their significance and biological relevance does not appear to be entirely clear [8,20,27,31,60]. Our findings of varying biological relevance of sperm SPs in the ejaculate strengthen the idea that a specific sub-populational structure, depicted by spermatozoa with different kinematic patterns, must be revised. Other authors have suggested some sperm SPs can be affected by a treatment [26] or by specific processes (e.g., freezing-thawing) indicating that some sperm SPs may have biological relevance [25] whereas others, co-existing in the same sample, may not [8,26,61,62]. Our Bayesian results indicated that SP1, SP2, and SP3 were different and that these differences were relevant. The biological relevance of these SPs may thus lead to an invalidation of the classical definition of the sperm cluster or subpopulation. Relevance may relate to fertility. The variability of spermatozoa in an ejaculate can correlate with the variability of fertility among males [63], and lesser motility can also associate with lower fertility rates [2]. If velocity is a principal component that explains the kinematic variance of sperm SPs, and velocity can relate to fertility, we thus need to further clarify the biological relevance of these SPs.

Sperm SPs should then be assessed using a criterion of utility in relation to biological importance. To our knowledge, this is the first study in which an assessment of statistically relevance of ejaculate SPs has been carried out. Distinction between species, and between breeds, should be taken into account and implemented in this type of analysis. Previous studies have considered these distinction and comparisons carried out between species with regards to kinematics [16] and morphology have identified differences [64]. The fact that there are differences between species suggest that new approaches may be required when managing the ejaculates. In any case, these differences must be relevant from the biological point of view and statistical differences may not be sufficient as criterion. There is thus a need to further examine the issue of biological relevance in the analysis of sperm SPs and to develop means to explore the biological meaning of data sets of clustered cells deriving from automated semen analyses.

## 5. Conclusions

Current approaches for the analysis of sperm kinematic SPs may not have a direct biological meaning and therefore, the biological relevance of sperm SPs needs to be reevaluated.

## Figures and Tables

**Table 1:** Kinematic variables (mean ± SEM) of the four sperm sub-populations (SPs) defined at 60 min post-thawing bull semen samples.

Sub-Population	SP_1_	SP_2_	SP_3_	SP_4_
Number of cells (%)	1300 (20.02)	1837 (28.29)	1243 (19.14)	2113 (32.54)
VCL	120.93 ± 1.2^a^	140.10 ± 1.02^b^	108.41 ± 1.23^c^	141.78 ± 0.95^b^
VSL	77.30 ± 1.12^a^	118.29 ± 0.94^b^	52.60 ± 1.14^c^	105.79 ± 0.87^d^
VAP	86.97 ± 1.01^a^	120.03 ± 0.85^b^	67.37 ± 1.03^c^	106.67 ± 0.79^d^
LIN	60.90 ± 0.58^a^	80.80 ± 0.49^b^	49.56 ± 0.59^c^	75.39 ± 0.46^d^
STR	82.05 ± 0.49^a^	92.73 ± 0.41^b^	77.37 ± 0.50^c^	95.72 ± 0.39^d^
WOB	70.36 ± 0.44^a^	83.75 ± 0.37^b^	62.42 ± 0.45^c^	76.87 ± 0.34^d^
ALH	3.71 ± 0.04^a^	3.28 ± 0.03^b^	3.89 ± 0.04^c^	3.93 ± 0.03^c^
BCF	9.17 ± 0.10^a^	8.97 ± 0.08^a^	8.13 ± 0.10^b^	11.32 ± 0.08^c^

SP_1_: medium velocity and linear-progressive; SP_2_: fast linear-progressive and oscillatory; SP_3_: slow nonlinear, non-progressive, and non-undulatory; SP_4_: fast linear-progressive and undulatory. Number of cells = 6493. VCL = curvilinear velocity (µm·s^−1^); VSL = straight-line velocity (µm·s^−1^); VAP = average path velocity (µm·s^−1^); LIN = linearity of forward progression (%); STR = straightness (%); WOB = wobble (%); ALH = amplitude of lateral head displacement (µm); BCF = beat-cross frequency (Hz). SEM = standard error of the mean. ^a-d^ Different letters indicate differences between sperm SPs. *p* < 0.05.

**Table 2:** Eigenvectors of principal components (PCs) * for kinematic variables of bull sperm in three commercial extenders.

Extender	OptiXcell^®^			Triladyl^®^			Tris-EY		
Variable	PC_1_	PC_2_	PC_3_	PC_1_	PC_2_	PC_3_	PC_1_	PC_2_	PC_3_
VCL		0.960			0.915			0.899	
VSL		0.737		0.819			0.728		
VAP		0.888		0.723			0.761	0.610	
LIN	0.931			0.977			0.827		
STR	0.641		0.621	0.737					0.844
WOB	0.899			0.909			0.942		
ALH	−0.646	0.624			0.912			0.884	
BCF			0.801			0.952			0.668
Var Exp	37.51	35.26	16.59	46.50	29.18	14.48	38.14	30.51	21.33

Var Exp: variance explained in each PC. Total variance explained: OptiXcell^®^ = 89.36%; Triladyl^®^ = 90.16%; Tris-EY = 89.98%. *Expresses the more important variables in each PC. Only eigenvectors >0.6 are presented. VCL: curvilinear velocity; VSL: straight-line velocity; VAP: average path velocity; LIN: linearity of forward progression; STR: straightness; WOB: wobble; ALH: amplitude of lateral head displacement; BCF: beat-cross frequency.

**Table 3:** Kinematic variables (means ± SEM) of motile bull sperm SPs in different commercial extenders.

Kinematic Variables	VCL	VSL	VAP	LIN	STR	WOB	ALH	BCF
OptiXcell^®^								
SP_1_	120.62 ± 1.5^aβ^	74.41 ± 1.35^aβ^	77.17 ± 1.32^aβ^	59.36 ± 0.67^aβ^	91.87 ± 0.53^aβ^	62.48 ± 0.57^aβ^	3.96 ± 0.05^aβ^	11.38 ± 0.13^aβ^
SP_2_	165.50 ± 1.20^bβ^	144.29 ± 1.07^bβ^	143.07 ± 1.04^bβ^	87.16 ± 0.53^bβ^	97.52 ± 0.42^bβ^	86.77 ± 0.45^bβ^	3.80 ± 0.04^aβ^	10.00 ± 0.10^bβ^
SP_3_	128.03 ± 1.95^cβ^	43.81 ± 1.74^cβ^	81.97 ± 1.70^aβ^	31.53 ± 0.86^cβ^	50.71 ± 0.68^cβ^	62.23 ± 0.73^cβ^	4.49 ± 0.06^bβ^	6.73 ± 0.17^cβ^
SP_4_	86.70 ± 1.36^dβ^	74.24 ± 1.22^aβ^	77.73 ± 1.19^aβ^	83.07 ± 0.60^dβ^	92.64 ± 0.48^aβ^	88.08 ± 0.51^dβ^	2.05 ± 0.05^cβ^	6.76 ± 0.12^cβ^
Triladyl^®^								
SP_1_	129.45 ± 1.20^aγ^	111.73 ± 1.19^aγ^	111.19 ± 1.02^aγ^	85.91 ± 0.61^aγ^	97.22 ± 0.66^aγ^	86.06 ± 0.47^aγ^	3.29 ± 0.04^aγ^	8.62 ± 0.11^aγ^
SP_2_	74.94 ± 1.57^bγ^	31.09 ± 1.57^bγ^	42.28 ± 1.35^bγ^	41.45 ± 0.80^bγ^	72.34 ± 0.87^bγ^	55.77 ± 0.62^bγ^	2.97 ± 0.06^bγ^	7.56 ± 0.15^bγ^
SP_3_	168.80 ± 1.27^cγ^	79.89 ± 1.56^cγ^	96.62 ± 1.34^cγ^	46.80 ± 0.80^cγ^	80.53 ± 0.87^cγ^	57.38 ± 0.62^bγ^	6.17 ± 0.06^cγ^	8.80 ± 0.15^aγ^
SP_4_	158.77 ± 1.10^dγ^	120.00 ± 1.09^dγ^	119.45 ± 0.94^dγ^	75.78 ± 0.56^dγ^	97.66 ± 0.61^aγ^	75.52 ± 0.43^cγ^	4.31 ± 0.04^dγ^	14.13 ± 0.10^cγ^
Tris-EY								
SP_1_	108.68 ± 1.64^aδ^	30.28 ± 1.65^aδ^	66.08 ± 1.54^aδ^	25.85 ± 0.88^aδ^	43.95 ± 0.62^aδ^	59.21 ± 0.78^aδ^	3.94 ± 0.06^aβ^	6.54 ± 0.17^aδ^
SP_2_	137.08 ± 1.07^bδ^	122.67 ± 1.07^bδ^	124.09 ± 1.00^bδ^	88.45 ± 0.57^bβ^	95.11 ± 0.40^bδ^	90.79 ± 0.51^bδ^	2.85 ± 0.04^bγ^	8.39 ± 0.11^bδ^
SP_3_	74.15 ± 1.14^cδ^	45.17 ± 1.15^cβ^	48.47 ± 1.07^cδ^	58.92 ± 0.61^cδ^	88.19 ± 0.43^cδ^	64.62 ± 0.54^cδ^	2.66 ± 0.04^cδ^	8.49 ± 0.12^bγ^
SP_4_	168.04 ± 0.97^dδ^	118.06 ± 0.97^dγ^	118.09 ± 0.90^dγ^	70.01 ± 0.52^dδ^	96.56 ± 0.37^dγ^	70.22 ± 0.46^dδ^	4.96 ± 0.03^dδ^	12.62 ± 0.10^cδ^

VCL = curvilinear velocity (µm·s^−1^); VSL = straight-line velocity (µm·s^−1^); VAP = average path velocity (µm·s^−1^); LIN = linearity of forward progression (%); STR = straightness (%); WOB = wobble (%); ALH = amplitude of lateral head displacement (µm); BCF = beat-cross frequency (Hz); SP: subpopulation; SEM = standard error of the mean. Number of cells = 6493. ^a-d^ Within the same column and extender, different superscripts indicate differences among sperm SPs. ^βγδ^ Within the same column and SP, different superscripts indicate differences among extenders. *p* < 0.05.

**Table 4:** Kinematic bull sperm variables and estimated marginal posterior distributions of differences between commercial extenders Tris-EY, Triladyl^®^ and OptiXcell^®^.

Variable	Mean ± r.s.d.	CV	*D* ^1^	HPD_95%_^2^	*P*_0_^3^	*P_R_* ^4^
VCL	127.92 ± 44.94	36.56				
D_1_−D_2_			−12.91	−34.03, 6.82	0.07	0.63
D_1_−D_3_			−2.36	−22.61, 16.83	0.40	0.91
D_2_−D_3_			10.55	−13.81, 35.41	0.86	0.30
VSL	4.47 ± 2.86	63.93				
D_1_−D_2_			0.06	−0.16, 0.27	0.72	0.00
D_1_−D_3_			0.08	−0.12, 0.27	0.79	0.00
D_2_−D_3_			0.02	−0.18, 0.26	0.58	0.00
VAP	86.95 ± 46.48	56.22				
D_1_−D_2_			−11.74	−34.32, 12.04	0.13	0.59
D_1_−D_3_			−8.35	−29.68, 11.32	0.19	0.71
D_2_−D_3_			3.39	−21.56, 27.76	0.62	0.18
LIN	4.43 ± 2.83	64.05				
D_1_−D_2_			−0.06	−0.29, 0.14	0.28	1.00
D_1_−D_3_			0.08	−0.12, 0.27	0.79	0.00
D_2_−D_3_			0.14	−0.08, 0.36	0.89	0.00
STR	93.81 ± 40.68	44.58				
D_1_−D_2_			−6.97	−21.37, 5.78	0.13	0.46
D_1_−D_3_			−6.37	−21.84, 7.48	0.17	0.50
D_2_−D_3_			0.61	−14.05, 15.91	0.53	0.20
WOB	4.60 ± 2.87	62.61				
D_1_−D_2_			0.08	−0.20, 0.35	0.75	0.00
D_1_−D_3_			0.03	−0.24, 0.29	0.62	0.00
D_2_−D_3_			−0.05	−0.35, 0.22	0.35	1.00
ALH	65.45 ± 23.77	37.43				
D_1_−D_2_			−3.59	−12.97, 4.72	0.19	0.22
D_1_−D_3_			−6.32	−15.92, 3.38	0.08	0.09
D_2_−D_3_			−2.73	−13.49, 7.03	0.25	0.28
BCF	4.14 ± 2.97	71.97				
D_1_−D_2_			−0.15	−0.56, 0.30	0.22	1.00
D_1_−D_3_			0.17	−0.23, 0.62	0.82	0.00
D_2_−D_3_			0.32	−0.16, 0.80	0.93	0.00

D_1_, Tris-EY; D_2_, Triladyl^®^; D_3_, OptiXcell^®^; ^1^*D*: mean of the marginal posterior distribution of the difference; ^2^HPD_95%_, highest posterior density region at 95%; ^3^*P*_0_, probability of the difference being greater than zero when *D* > 0 and probability of the difference being lower than zero when *D* < 0; ^4^*P_R_*, probability of the difference being greater than R when *D* > 0 and less than R when *D* < 0. Number of cells = 6493. r.s.d.: residual standard deviation. CV: coefficient of variation (%). VCL = curvilinear velocity (µm·s^−1^); VSL = straight-line velocity (µm·s^−1^); VAP = average path velocity (µm·s^−1^); LIN = linearity of forward progression (%); STR = straightness (%); WOB = wobble (%); ALH = amplitude of lateral head displacement (µm); BCF = beat-cross frequency (Hz).

**Table 5:** Descriptive parameters and features of the estimated marginal posterior distributions of differences for kinematic bull sperm variables between *Bos taurus* and *Bos indicus*.

	Mean ± r.s.d.	CV	*D* ^1^	HPD_95%_^2^	*P*_0_^3^	R^4^	*P_R_* ^5^
VCL	126.56 ± 44.93	36.54	11.65	−3.16, 26.55	0.95	15.00	0.30
VSL	4.47 ± 2.86	64.06	0.03	−0.13, 0.20	0.66	15.53	0.00
VAP	85.61 ± 46.48	56.91	8.57	−10.70, 9.55	0.84	13.57	0.26
LIN	4.42 ± 2.83	64.15	0.10	−0.06, 0.27	0.88	7.94	0.00
STR	93.00 ± 40.69	45.03	2.13	−10.37, 14.89	0.64	6.34	0.22
WOB	4.58 ± 2.87	62.82	0.02	−0.19, 0.23	0.60	5.79	0.00
ALH	65.09 ± 23.76	37.82	−0.16	−8.71, 7.49	0.46	−0.49	0.52
BCF	4.13 ± 2.97	72.15	0.18	−0.18, 0.56	0.85	1.23	0.00

^1^*D*: mean of the marginal posterior distribution of the difference B*t*-B*i*, where B*t* is *Bos taurus* and B*i* is *Bos indicus*; ^2^HPD_95%_, highest posterior density region at 95%; ^3^*P*_0_, probability of the difference being greater than zero when *D* > 0 and probability of the difference being lower than zero when *D* < 0; ^4^R, relevant value estimated as 1/3 standard deviation; ^5^*P_R_*, probability of the difference being greater than R when *D* > 0 and less than R when *D* < 0. Number of cells = 6493. r.s.d.: residual standard deviation. CV: coefficient of variation (%). VCL = curvilinear velocity (µm·s^−1^); VSL = straight-line velocity (µm·s^−1^); VAP = average path velocity (µm·s^−1^); LIN = linearity of forward progression (%); STR = straightness (%); WOB = wobble (%); ALH = amplitude of lateral head displacement (µm); BCF = beat-cross frequency (Hz).

**Table 6:** Kinematic bull sperm variables and estimated marginal posterior distribution of differences between motile sperm SPs.

Variable	Mean ± r.s.d.	CV	*D* ^1^	HPD_95%_^2^	*P*_0_^3^	*P_R_* ^4^
VCL	125.80 ± 42.89	35.32				
SP_1_−SP_2_			−21.49	−24.78, −18.36	0.00	0.00
SP_1_−SP_3_			11.92	8.52, 15.28	1.00	0.04
SP_1_−SP_4_			−20.72	−23.77, −17.69	0.00	0.00
SP_2_−SP_3_			33.41	30.22, 36.42	1.00	1.00
SP_2_−SP_4_			0.77	−2.09, 3.39	0.70	0.00
SP_3_−SP_4_			−32.64	−35.67, −29.59	0.00	0.00
VSL	4.47 ± 2.86	64.01				
SP_1_−SP_2_			−0.10	−0.31, 0.10	0.18	1.00
SP_1_−SP_3_			−0.04	−0.27, 0.17	0.35	1.00
SP_1_−SP_4_			−0.02	−0.21, 0.18	0.44	1.00
SP_2_−SP_3_			0.05	−0.15, 0.26	0.69	0.00
SP_2_−SP_4_			0.08	−0.10, 0.26	0.80	0.00
SP_3_−SP_4_			0.03	−0.17, 0.22	0.59	0.00
VAP	83.35 ± 39.79	49.71				
SP_1_−SP_2_			−41.54	−44.49, −38.55	0.00	0.00
SP_1_−SP_3_			24.36	21.05, 27.47	1.00	1.00
SP_1_−SP_4_			−27.97	−30.99, −25.18	0.00	0.00
SP_2_−SP_3_			65.89	62.87, 68.75	1.00	1.00
SP_2_−SP_4_			13.57	10.91, 16.01	1.00	0.49
SP_3_−SP_4_			−52.32	−55.01, −49.32	0.00	0.00
LIN	4.45 ± 2.83	63.72				
SP_1_−SP_2_			−0.05	−0.26, 0.14	0.31	1.00
SP_1_−SP_3_			−0.10	−0.33, 0.11	0.18	1.00
SP_1_−SP_4_			0.07	−0.11, 0.27	0.78	0.00
SP_2_−SP_3_			−0.05	−0.26, 0.15	0.31	1.00
SP_2_−SP_4_			0.12	−0.05, 0.30	0.92	0.00
SP_3_−SP_4_			0.18	−0.02, 0.38	0.96	0.00
STR	90.76 ± 36.09	40.54				
SP_1_−SP_2_			−33.46	−36.10, −30.82	0.00	0.00
SP_1_−SP_3_			19.10	16.19, 21.90	1.00	1.00
SP_1_−SP_4_			−19.89	−22.46, −17.33	0.00	0.00
SP_2_−SP_3_			52.55	49.95, 55.23	1.00	1.00
SP_2_−SP_4_			13.56	11.13, 15.88	1.00	1.00
SP_3_−SP_4_			−38.99	−41.68, −36.60	0.00	0.00
WOB	4.59 ± 2.87	62.64				
SP_1_−SP_2_			0.08	−0.13, 0.28	0.77	0.00
SP_1_−SP_3_			0.04	−0.18, 0.27	0.64	0.00
SP_1_−SP_4_			0.06	−0.13, 0.27	0.74	0.00
SP_2_−SP_3_			−0.03	−0.24, 0.17	0.37	1.00
SP_2_−SP_4_			−0.01	−0.20, 0.17	0.44	1.00
SP_3_−SP_4_			0.02	−0.18, 0.23	0.58	0.00
ALH	63.52 ± 20.85	33.42				
SP_1_−SP_2_			−19.84	−21.34, −18.26	0.00	0.00
SP_1_−SP_3_			10.78	9.05, 12.39	1.00	1.00
SP_1_−SP_4_			−14.73	−16.27, −13.25	0.00	0.00
SP_2_−SP_3_			30.62	29.09, 32.16	1.00	1.00
SP_2_−SP_4_			5.11	3.82, 6.42	1.00	1.00
SP_3_−SP_4_			−25.51	−26.96, −24.03	0.00	0.00
BCF	4.16 ± 2.96	71.49				
SP_1_−SP_2_			0.32	0.11, 0.53	1.00	0.00
SP_1_−SP_3_			0.01	−0.22, 0.24	0.53	0.00
SP_1_−SP_4_			0.03	−0.18, 0.23	0.62	0.00
SP_2_−SP_3_			−0.31	−0.54, −0.10	0.00	1.00
SP_2_−SP_4_			−0.29	−0.47, −0.09	0.00	1.00
SP_3_−SP_4_			0.02	−0.18, 0.23	0.58	0.00

^1^*D*: mean of the marginal posterior distribution of the difference between sperm subpopulations: SP_1_, SP_2_, SP_3_, and SP_4_; ^2^HPD_95%_, highest posterior density region at 95%; ^3^*P*_0_, probability of the difference being greater than zero when *D* > 0 and probability of the difference being lower than zero when *D* < 0; ^4^*P_R_*, probability of the difference being greater than R when *D* > 0 and less than R when *D* < 0. Number of cells = 6493. r.s.d.: residual standard deviation. CV: coefficient of variation (%). VCL = curvilinear velocity (µm·s^−1^); VSL = straight-line velocity (µm·s^−1^); VAP = average path velocity (µm·s^−1^); LIN = linearity of forward progression (%); STR = straightness (%); WOB = wobble (%); ALH = amplitude of lateral head displacement (µm); BCF = beat-cross frequency (Hz).

**Table 7:** Percentage of sperm cells from bulls *Bos taurus* and *Bos indicus* in each kinematic subpopulation characterized in semen diluted in three commercial extenders.

	SP_1_	SP_2_	SP_3_	SP_4_
Extender				
OptiXcell^®^	22.56^aα^	36.06^bα^	13.60^cα^	27.78^dα^
Triladyl^®^	29.83^aβ^	17.25^bβ^	17.31^bβ^	35.62^dβ^
Tris-EY	12.00^aγ^	28.47^bγ^	24.87^cγ^	34.66^dγ^
Species				
*Bos taurus*	23.13^aδ^	35.82^bδ^	14.61^cδ^	26.43^dδ^
*Bos indicus*	18.20^aε^	23.89^bε^	21.79^cε^	36.12^dε^

Each row indicates the percentage of spermatozoa in each sperm subpopulation. Cluster; sum of percentage for each extender = 100, and species = 100. Total number of cells for each extender: OptiXcell^®^ = 2666, Triladyl^®^ = 1606, Tris-EY = 2221. Total number of cells for each species: *Bos taurus* = 2395, *Bos indicus* = 4098. ^a, b, c, d^ Superscript indicates differences within row regarding sperm subpopulation. ^α, β, γ^ Superscript indicates differences within column for each extender; ^δ, ε^ Superscript indicates differences within column for each species; chi squared (χ^2^) test, *p* < 0.05.
